# An Update on Cephenemyiosis in the European Roe Deer: Emergent Myiasis in Spain

**DOI:** 10.3390/ani11123382

**Published:** 2021-11-26

**Authors:** Patrocinio Morrondo, Gerardo Pajares, María Sol Arias, Néstor Martínez-Calabuig, Susana Remesar, David García-Dios, Pablo Díaz, Ceferino Manuel López, Rosario Panadero, Pablo Díez-Baños

**Affiliations:** INVESAGA Group, Department of Animal Pathology, Faculty of Veterinary, University Santiago de Compostela, 27002 Lugo, Spain; patrocinio.morrondo@usc.es (P.M.); gerardopajaresbdequiros@gmail.com (G.P.); mariasol.arias@usc.es (M.S.A.); nestor.martinez@rai.usc.es (N.M.-C.); susana.remesar@usc.es (S.R.); david.garcia@rai.usc.es (D.G.-D.); pablo.diaz@usc.es (P.D.); c.lopez@usc.es (C.M.L.); pablo.diez@usc.es (P.D.-B.)

**Keywords:** roe deer, *Cephenemyia stimulator*, myiasis, Europe, Spain

## Abstract

**Simple Summary:**

Cephenemyiosis is a myiasis developing in the nasal cavity and pharynx of roe deer; it is widely spread in the range of distribution of this ungulate in Europe. In the last decade, this myiasis is expanding its distribution along its southernmost and northernmost range margins in Europe; thus, it is of the utmost importance to unravel all the epidemiological and clinical aspects of this myiasis, as well as implementing surveillance measures including reliable and non-invasive diagnostic techniques to monitor its expansion and adaptation to different ecosystems and/or hosts and to reduce the negative impact on roe deer populations.

**Abstract:**

*Cephenemyia stimulator* is a Palearctic species developing in the nasal cavity and pharynx of roe deer (*Capreolus capreolus*). It is widely spread in the range of distribution of this ungulate in Europe. Since the first report of *C. stimulator* in Spain in 2001, a rapid geographic expansion has been observed, first in the north of the country, with high prevalence and intensities of infestation that caused some mortal cases, and, lately, also in Extremadura and Andalucía, the southernmost populations of European roe deer. These observations suggest an adaptation of this parasite to different ecosystems of the Iberian Peninsula. Almost simultaneously, *C. stimulator* is also expanding its range to northern Europe, with the first cases being reported in Sweden. Thus, *Cephenemyia stimulator* may be an example of a parasite currently displaying distributional changes along its southernmost and northernmost range margins. Thus, it is of the utmost importance to unravel all the epidemiological and clinical aspects of this myiasis, as well as implementing surveillance measures including reliable and non-invasive diagnostic techniques to monitor its expansion and adaptation to different ecosystems and/or hosts and to reduce the negative impact on roe deer populations.

## 1. Introduction

Nasal bots are obligatory myiasis-causing larvae belonging to the subfamily Oestrinae (Family Oestridae), which includes several important genera infecting Cervidae (*Cephenemyia* and *Pharyngomyia*), other Artiodactyla species (*Oestrus*, *Kirkioestrus* and *Gedoelstia*), horses (*Rhinoestrus*), camels (*Cephalopina*), African elephants (*Pharyngobolus*) and Australian kangaroos (*Tracheomyia*) [1].

*Cephenemyia* spp. develops in the nasal and pharyngeal cavities of different species of Cervinae and Odocoileinae [2]. In this respect, the genus *Cephenemyia* is considered quite host-specific and rarely parasitizes unsuitable hosts [1,3].

Eight species in the genus *Cephenemyia* can infect cervids (Table 1); these species are confined to either the Neartic (new world) or Paleartic (old world) area excepting *Cephenemyia trompe*, which is distributed throughout the northern Holarctic region. Among them, *Cephenemyia stimulator* is a species specific to roe deer that is widely spread in the range of distribution of this ungulate in Europe [4].

## 2. Morphology of Flies and Larvae

The imagos of the different species of *Cephenemyia* are very similar and have a particular disposition of yellow and black hair. However, since the coloration varies among species, other features should be considered for achieving a reliable morphological identification [5].

The body of *C. stimulator* flies is covered with yellow and orange hairs, sometime acquiring a slightly reddish hue similar to that of bumblebees (*Bombus* spp). This coloration pattern is an adaptation for protecting themselves from possible predators by adopting a shape and color mimicking that of poisonous species [6]; moreover, they are fast flyers (calyptrates). The head is large in comparison to the rest of the body, with large eyes and short antennae [5,6,7]. Males have the phalosome covered with thin spines.

*C. stimulator* adults, such as other oestrid flies, do not feed during the adult stage, so they depend on an efficient assimilation and storage of nutrients during their parasitic larval stage [6]. Adults do not have functional mouthparts and their large eyes enable the localization of potential hosts, as well as suitable mates. Adults have a short lifespan and females emerge from the puparium with fully developed eggs ready for fertilization.

The three larval stages of *C. stimulator* are elongated and have twelve segments with the ventral region flatter than the dorsal one [8]. The first segment, the cephalic, is mobile and can be retracted within the first thoracic segment (segment II). They are covered with spines that, together with the large mouth hooks, serve to attach to the mucosa of the host, thus avoiding being expelled by the host’s defense mechanisms, such as cough and sneezing [9].

**Figure 1:**
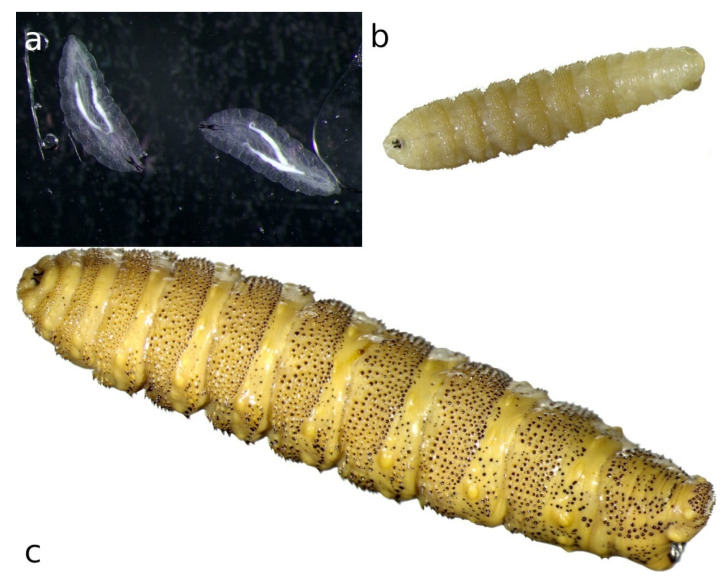
(**a**) First, (**b**) second and (**c**) third larval stages of *Cephenemyia stimulator*.

Larvae can be differentiated in three development stages according to their total length and color of the cuticle, as well as the shape and color of the posterior peritremes [10]. The first stage larvae (L1) were first described by Ullrich in 1936 [11] and more recently by Quintela [10]; they are small (2.27 ± 0.80 mm), white and flattened dorsoventrally (Figure 1a). The segments are surrounded by spines and both the dorsal and ventral areas of segments II and XII have numerous rows of denticles allowing researchers to differentiate this species from *C. trompe*, which only has rudimentary denticles in the dorsal part. In addition, the spines are more evident on the ventral and lateral surface of the thoracic and abdominal segments [12]. The posterior peritremes are cloverleaf-shaped, dark brown-colored, with small unpigmented peritremal buttons and scarce and large respiratory pores [10].

Second stage larvae (L2) are yellowish and their length ranges between 3 and 13 mm (average 10.03 ± 3.10) (Figure 1b). They are flattened dorsoventrally, similar to the rest of the larval stages; the dorsal surface is rounded and, in a sagittal cut, it has an ovoid appearance. The distribution of the spicules is similar to that of L1, but with a lower density in the dorsal region [11]; in addition, the X and XI segments lack spines, or have up to four rows of spines. Different authors pointed out that the number of spines on the dorsal face is higher than that on the ventral one and that, from the III to the XI segment, they present between five and eight rows of spines disposed in an irregular form [5,6,13].

**Figure 2:**
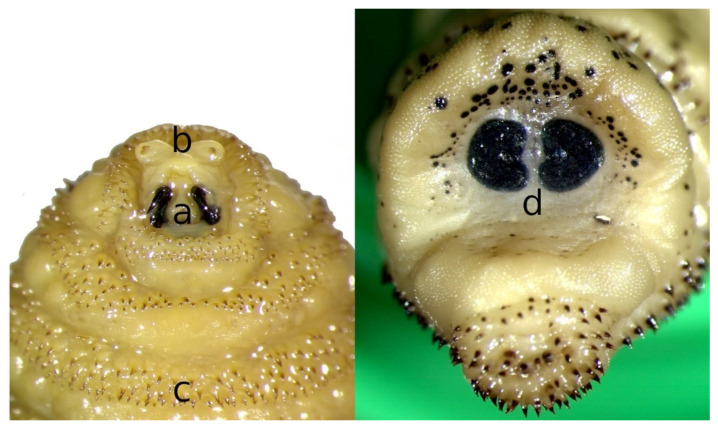
Morphological details of *Cephenemyia stimulator* third instar larvae: (**a**) mouth hooks, (**b**) antennal lobes, (**c**) cuticular spines and (**d**) posterior peritremes.

Third stage larvae (L3) can reach 30 mm in length with an average of 24.73 ± 5.94 mm (Figure 1c). The posterior peritremes are reniform. Its shape and color are similar to those of L2 but the amount of melanin increases with maturation, leading to a darkening of the cuticle and the spines; dark spots appear on the body of mature larvae, especially in the last segment. Zumpt [5] indicated that the dorsal part of segment XI has several medial spines. The main features to be considered for the identification of L3 of *C. stimulator* are the shape and arrangement of the antennal segments (Figure 2a), the size of the mouth hooks (Figure 2b), the distribution pattern of the cuticular spines (Figure 2c) and the shape of the posterior peritremes (Figure 2d) [5,10].

The general morphology and arrangement of the digestive and excretory systems of *C. stimulator* L3 are similar to those of *O. ovis* [14]. Micro-computed tomography revealed that the digestive system shows a uniformly narrow midgut that lacks gastric caeca and forms six loops before the beginning of the hindgut. The hindgut first runs anteriorly up to the fourth/fifth abdominal segment, where it turns and runs dorsally towards the posterior end of the body, opening into the anus. The two salivary glands are elongated, connected at their anterior ends by the common salivary duct, forming a ’glandular band’ by coming together at their posterior ends. The excretory system consists of two pairs of tubules that open into the transition between midgut and hindgut. The L3 of *C. stimulator* has a high abundance of fat body cells arranged in a kind of sheet that covers both sides and much of the dorsal aspect of the block formed by the digestive and excretory organs [14].

Finally, pupae of *C. stimulator* are from 16 to 20 mm long and present a black chitinous coating protecting the imago.

## 3. Life Cycle and Chronobiology

**Scheme 1:**
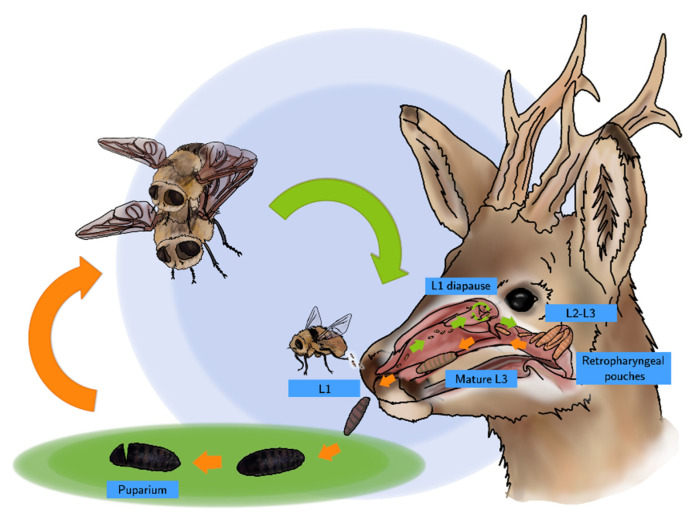
Life cycle of *C. stimulator*.

A new life cycle begins after copulation (Scheme 1), when fertilized females of *C. stimulator* detect a roe deer attracted by its odor or by the CO_2_ exhaled. Females can contain up to 500 larvae and, during their lifespan (16 days), they deposit or eject packets of 30–50 L1s in the nostrils of different specimens of roe deer. This strategy enables a higher survival rate, since a number of roe deer, with different age and immune status, can be infested; in addition, competition among larvae is lower [6,7,15]. L1s are surrounded by a thick, gelatinous fluid that promotes their adhesion and protects them from desiccation.

**Figure 3:**
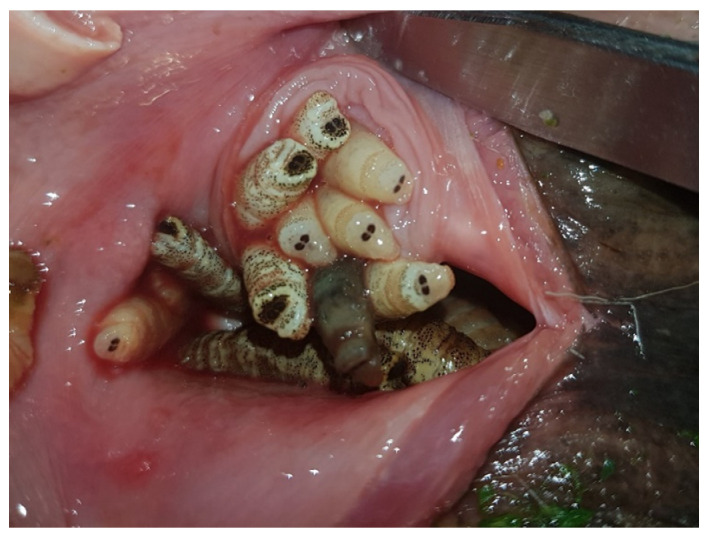
*C. stimulator* larvae in the retropharyngeal pouches of a roe deer (ventral view).

The endogenous life cycle begins when the L1 moves into the nose or mouth towards the nasal cavities aided by their hooks and spines which constitute a defense mechanism against the host’s attempts to get rid of them through coughs, sneezes and sudden movements of the head. L1s can enter in a phase of hypobiosis or diapause as an adaptive response to adverse weather conditions; thus, their full development does not take place until environmental conditions become favorable for their survival. Diapause can occur at any stage of development, but usually occurs in the L1 or pupal phase [1,6,15]. Once L2 have been developed, they go towards the choanas, pharynx and larynx, the preferred location of L2s and L3s. In a number of roe deer, these larvae invade the retropharyngeal recesses that distend into “pouches” or diverticula harboring up to 30 larvae (Figure 3). In roe deer heavily infested by *C. stimulator*, larvae are grouped in the pharyngeal recesses as a cluster; the anterior part of the larva is fixed inside the foseta and the posterior end is oriented towards the opening of the recess. Paired retropharyngeal recesses were the preferred sites for the growing of both L2s and L3s of *Cephenemyia*, *Cephalopina* and *Rhinoestrus* [1].

Once their growth is complete, mature L3s progress to the upper airways following an inverse process to that of the L1s and L2s. After reaching the nostrils, L3s exit the host aided by roe deer coughing or sneezing, or they simply crawl out themselves [6,15,16]. In the environment, L3s bury into the ground among the leaf litter since they are lucifuge and, finally, they pupate. Inside the puparium, L3s result in females and males of *C. stimulator* that emerge after about 2–3 weeks, as long as the weather conditions are favorable [6,7,15], starting a new cycle.

Regarding the chronobiology, mating and host-seeking activities of all species of oestrids occur on warm and sunny days, with temperatures between 20 and 30 °C [17]. It has been reported that the seasonal dynamics of *C. stimulator* depend directly on the climatic conditions that determine the geographical distribution and the chronobiology of this myiasis in each region [6,18].

Adults of *C. stimulator* are active from late May to mid-September, when the temperature exceeds 13 °C [18] and their activity reduces under lower temperatures or with precipitation [19].

Vegetation cover also seems to influence the intensity of infestation, as this is higher in areas with lower density of trees [20]. However, between 200 and 1000 m of altitude, this does not seem to influence the viability of *C. stimulator* [18].

The chronobiology of this myiasis has been described in several European countries. In roe deer from Poland, larvae of *C. stimulator* were observed throughout the year, with L2s predominating between April and July and L3s from April to August [21]. Similar observations were reported in Hungary, where roe deer harbored L1s from late July to April–May of the following year, many of them in diapause, L2s between April and May and L3s between April and August [4].

**Scheme 2:**
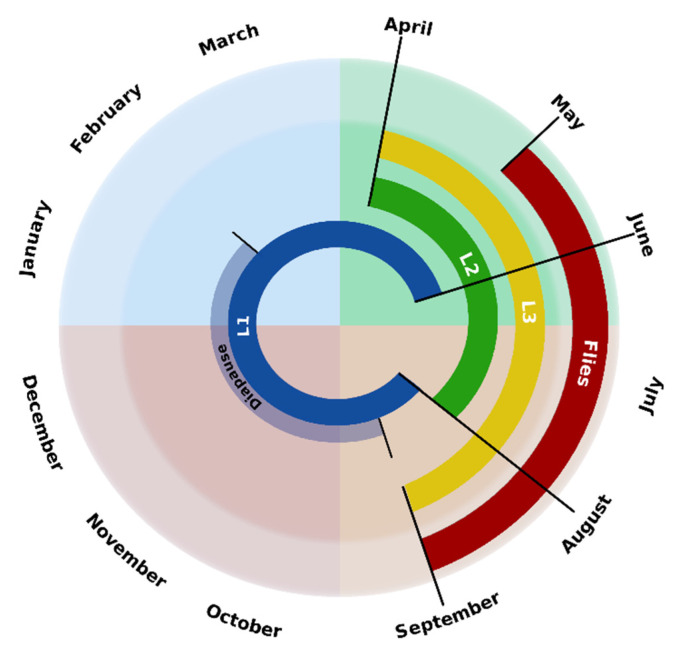
Chronobiology of *C. stimulator* in northwestern Spain.

According to the climatic conditions (temperature, precipitation, insolation, etc.) of northwestern Spain, as well as the observation of the different larval stages in roe deer by different authors [7,13,15,22], the chronobiology of *C. stimulator* in this area is that represented in Scheme 2.

Between May and September, the days have a remarkable number of hours of light, there is absence of strong wind gusts and average temperatures range between 15 and 20 °C, being the most favorable conditions for the flight of the flies. From September to February the average temperatures range between 9 and 16 °C, registering minimum temperatures of 2–3 °C; therefore, in these months, there is larval diapause and only L1s are found. However, the temperatures recorded between April and July (averages from 12 to 20 °C) favor the development of the larvae, noting that, in this period, the active phase of the endogenous cycle occurs, since, in the roe deer, the three larval stages have been found. Finally, considering that, in April, L3s can already be observed and that the pupation period in the oestrids lasts 20–30 days, the first flies may be observed in May and, as in other Oestridae, their activity lasts throughout the summer.

Finding different instars of *C. stimulator* simultaneously in the same host could be explained by the production of several larval generations per year [20], successive reinfestations, the ability of first-instar larvae to become hypobiotic and overwinter into the host and/or also by an asynchronous and gradual development of the larvae parasitizing the same host [2].

## 4. Epidemiology

*C. stimulator* is widely spread in the range of distribution of roe deer in Europe. As occurs with other Oestridae, several factors influencing the prevalence and intensity of infestation have also been identified for *C. stimulator*.

### 4.1. Environment and Climate

Distribution, prevalence and intensity of cephenemyiosis depend on the interaction among host density, environment and climate, with the latter being mainly related to the timing and duration of off-host periods [23]. In roe deer from Hungary, higher prevalence and mean intensity of *C. stimulator* were consistent with higher population densities and lower forest cover [20].

It has been stated that climate change will modify the geographical distribution and abundance of several parasite species [24,25]. In Spain, at the southern range margin of distribution of roe deer, *C. stimulator* was first reported in 2001 and affected one roe deer from Ciudad Real (Central Spain) that had been imported from France [26]. After this citation, a rapid expansion has been observed, especially in the north of the country. Thus, in northwestern Spain, this parasite was found for the first time in 2005 and a rapid increase in prevalence and intensity, together with some mortal cases, was registered afterwards [27]. In the last decade, this species was also reported parasitizing roe deer in northeast and southern Spain [7,28]. This rapid spread suggests an adaptation of this species to different ecosystems of the Iberian Peninsula. Almost simultaneously, *C. stimulator* was also expanding its distribution in northern Europe, with the first cases having been reported in Sweden in 2013 [29]. Thus, *C. stimulator* may be an example of a parasite currently displaying distributional changes along its southernmost and northernmost range margins.

### 4.2. Gender

It has been reported that *C. stimulator* prevalence and intensity are higher in bucks than does [21]. Thus, in different studies conducted in northwest Spain on hunted killed roe deer, the prevalence was always higher in males (45,5–55% vs. 14–25%). Similarly, in a recent investigation performed in the same region on roe deer dead by accident or natural causes, males were 4.65 times more likely to be infested than females [13].

In contrast, in sport-hunted roe deer from Hungary, 34.60% of the bucks killed between mid-April and September were infected, being this percentage of infection lower than that found in does (43.50%) that were killed from October to February [20]. However, intensity was significantly higher in bucks (8.87 larvae/animal) than in does (5.94 larvae/animal). The different hunting periods for bucks and does could be responsible for the lower prevalence in the former, as they were harvested mainly in summer months, which coincides in between two consecutive parasite generations.

The territorial behavior of roe deer bucks may favor the encounter with bot flies, because they are often forced to leave the forest and wander in open spaces where they are more vulnerable to larviposition [30].

### 4.3. Age

In general, there is strong evidence that prevalence increases with age. In this sense, a higher prevalence of infestation by *C. stimulator* was found in adult roe deer (62%) from NW Spain than in young animals (23%) [6]. This difference has been attributed to the fact that fawns, especially during the first months of life, spend much time hidden among the vegetation and protected from gravid flies [6,13,31], which are active between April to September.

However, in Hungary, prevalence and mean intensity of infestation were significantly higher in fawns (54.9%) than bucks (34.6%) and does (43.5) [20].

A negative correlation between the age and the intensity of infestation has been widely recorded. Rolandsen et al. [23] found higher parasite burdens in moose fawns and yearlings than in adults and Király and Egri [20] observed that roe deer fawns were more intensely infested (24.50 larvae/animal) than adults (8.87 bucks; 5.94 does). Intensity was also higher in young (23.3 ± 23.7 larvae/animal) than in adult roe deer (14.6 ± 15.9 larvae/animal) from NW Spain [6]. In other oestrids, such as *Hypoderma*, a partially protective immune response is developed after infection, causing the destruction of a high proportion of larvae in reinfested animals [32]; alternatively, a less efficient defensive behavior in fawns against fly harassment could favor their infestation [20].

### 4.4. Body Condition

It was reported that roe deer in poor body condition presented 10.7-fold higher risk of being infested by *Cephenemyia* than roe deer in good body condition [33]. In the case of roe deer bucks, the territorial behavior leads to intense energy wasting; therefore, it is difficult to determine whether the loss of body condition is the cause or the consequence of the infestation [20].

The deterioration of the body condition may be accelerated because individuals in poor conditions are unable to control parasitic infections [34] by producing an adequate immune adaptive response that protects them against reinfestations [35,36]. Nevertheless, it was demonstrated that low infestations with 6–11 larvae of *C. stimulator* did not affect roe deer condition [37].

## 5. Clinical Signs

*Cephenemyia* gravid flies approaching roe deer cause stress, negatively interfering with food intake [38]. In endemic areas, behavioral changes to avoid infestation have been observed in roe deer; in fact, they avoid open spaces at hours of maximum flight activity and feed in areas with greater vegetation cover [39]. In addition, ungulates prevent the larviposition around their nostrils by lowering and shaking their heads, sneezing, barking and even undertaking long runs and jumps [40].

The physical narrowing of the upper airways due to larval presence is the major cause of clinical signs. *C. stimulator* larvae use their mouth hooks to fixate and make their way through the tissues. Activity of mouth hooks and segment spines, together with the production of abundant nitric oxide, lead to a significant irritation of the nasal mucosa, eroding it [41]. Thus, few weeks after larviposition, nasal discharge and sneezing become evident and frequent. Sometimes, these lesions are complicated by secondary infections and it has been reported that a high number of L1s results in the existence of purulent exudates leading to respiratory complications or to secondary fly attacks [13]. Therefore, affected animals breathe through the mouth interfering with grazing and rumination, as observed in sheep with oestrosis [1].

Other signs related to larval stages of *C. stimulator* are coughing, sinusitis, respiratory stridor mainly on inspiration, nasal discharge possibly with mild epistaxis, exercise intolerance and open mouth breathing. Pulmonary auscultation is usually normal but is complicated by sounds referred to the upper airway [7]. The large size of *C. stimulator* L3s leads to an increase in volume of the retropharyngeal sacs, with manifest histological alterations. The internal aspect of the recess is mottled, rough and cracked. Histologically, loss of epithelium, degenerative changes and squamous metaplasia of the nasal and olfactory areas are observed; in addition, there is a submucosal oedema with infiltration of leukocytes, especially eosinophils [42,43]. Occasionally, *C. stimulator* larvae located in the pharyngeal recesses are suctioned into the lung, provoking pneumonia that can be fatal [9,44,45].

The severity of the disease is related to the larval burden; thus, a moderate number of larvae (<30 L3) is generally well tolerated by the animals, whereas 30–80 L3s have a negative impact on their survival [46]. Heavily infested animals present a poor body condition, weakness, apathy and low vitality [7,18,23].

## 6. Diagnosis

The diagnosis of cephenemyiosis can be based on clinical signs, such as constant and explosive sneezing, alertness with continuous expulsion of air, dilated nostrils, scratching and tapping of the nasal area with the legs, runs for no apparent reason and, in general, state of nervousness. However, these signs are nonspecific and very difficult to observe in wild animals such as roe deer [6].

Post mortem examination is the gold standard for the diagnosis of cephenemyiosis; nevertheless, it is mostly restricted to hunting periods or to sporadic findings of dead animals. For these reasons, it is difficult to obtain information throughout the year [6]. Reliable data on the prevalence and intensity of *C. stimulator* infestation can only be obtained after a thorough examination of the nasal and pharyngeal cavities of roe deer. Most larvae are in the nasal turbinate (46.9%) and the retropharyngeal sacs (35.8%) and, less frequently, in the glottis (14.7%), mouth (1.5%) and trachea (1.1%) [13]. Due to the small size of L1s, nasal cavity and pharynx should be thoroughly washed with pressurized water to drag the first instars, that are subsequently retained in a sieve with a mesh size of 150 µm. Finally, L1s are collected directly from the sieve under a stereomicroscope.

In live animals, a definitive diagnosis of *Cephenemyia* infestation can be achieved by endoscopic examination, allowing larvae to be detected, often found within the retropharyngeal pouches. These pouches may also be seen radiographically [47]. Computed tomography has also proved to be effective and safe for the manipulation and study of living animals [46]. However, all these techniques are not appropriate for routine diagnosis in wild animals because they are expensive and not applicable under field conditions; in addition, they are stressful for roe deer since they require their capture and immobilization.

As regards the immunodiagnosis of *C. stimulator* in roe deer, there are studies suggesting the usefulness of indirect ELISA [22,48,49]. Some trials using somatic antigens of *C. stimulator* L3s and a non-specific immunoconjugate proved that up to 38% of the supposed negative cases (without finding post mortem larvae) were seropositive [48,49]. It has been suggested that these results are related to L1 infestations, since these larvae are small and difficult to observe, as well as to the presence of specific antibodies that could persist beyond the elimination of any larval stage.

Arias et al. [22] tested two antigen complexes obtained from L2s, including *C. stimulator*, excretory/secretory antigens (CsES) and *C. stimulator* somatic antigens (CsSA). In addition, the composition of each antigen was analyzed using an electrophoresis system. CsSA showed four exclusive bands (17–19, 62, 65 and 67–70 kDa). A positive correlation between immunoglobulin G (IgG) values and total number of larvae was found with both CsES and CsSA. The highest sensitivity value, negative predictive value and negative likelihood ratio were obtained using CsES. The highest specificity value, positive likelihood ratio and kappa value were achieved with CsSA. The predictive values of ELISA using CsES and CsSA reached statistical significance and seroprevalence values were 26–44%. All these results suggest that the use of ELISA with CsES and CsSA can be a promising tool for the non-invasive diagnosis of *Cephenemyia* infestation in roe deer.

## 7. Prevalence and Infestation Intensity

Several studies, summarized in Table 2, have dealt with the prevalence and intensity of infection by *C. stimulator* in European roe deer. It should be noted that prevalence values greatly vary depending on the method employed (necropsy or ELISA) and the area of study.

Most of the studies have used necropsy to investigate the presence of cephenemyiosis. This myiasis has been well studied in central Europe, especially in Hungary [20,50,51,52,53,54] and Czech Republic [18,55,56], with varying results in prevalence and intensities of infestation. Extensive data from northern Spain are also available [13,30,31], showing prevalence ranging from 31.6% to 43.2% and mean parasite burdens of 16.9–19.7 larvae/animal; these data indicate that cephenemyiosis is a recent myiasis that is well established in roe deer from that area.

In contrast, seroprevalence studies of *C. stimulator* are still scarce in Europe. In France, a seroprevalence of 32 and 43.2% was found in 1998 and 1999, respectively [49]. In Spain, Arias et al. [31] analyzed blood samples from northern populations of roe deer for two decades (1994–2014). As expected, over the period 1994–2000, no seropositive roe deer was detected by ELISA, since the first report dates 2001 [26]. However, 38% (CI 35–42) of roe deer hunted between 2007 and 2014 showed *C. stimulator* antibodies. It is worth noting that the presence of specific antibodies indicates exposure to the parasite but not necessarily an active infestation. Seroprevalence varied significantly from year to year and oscillated between 36% (28–43) in 2007 and 60% (51–69) in 2014. When comparing the necropsy findings to immunodiagnosis, a positive and significant correlation between the seroprevalence values and both the number of animals harboring *C. stimulator* larvae and the mean intensity of infestation was observed. The combined use of direct and indirect techniques demonstrated a high prevalence of *C. stimulator* in roe deer in the northwest of Spain, which certainly highlights the importance of this myiasis during the last years.

## 8. Treatment and Control

The administration of antiparasitic drugs in free-living wildlife is a widely discussed issue, with obvious ethical implications. In general, wildlife diseases should be managed from an ecological perspective, with an understanding of the role that disease plays in the ecosystems. However, concerns about the impact of diseases on the welfare on individual animals may drive a need for managing wildlife diseases.

Macrocyclic lactones have been widely proven to be effective against ectoparasites. Not surprisingly, most investigations on the therapeutic efficacy of different macrocyclic lactones against oestrid larvae have been performed in domestic ruminants [60,61]. In contrast, such trials in wildlife are scarce and limited to animals in captivity under controlled conditions, avoiding a reliable determination of the pharmacokinetics of these drugs [62].

The administration of ivermectin to wild ruminants is normally applied in-feed [63]. However, its metabolism in the rumen reduces its bioavailability by 75% in comparison to parenteral application, which is severely limited in wild animals for practical reasons [64].

In roe deer, the experience on the use and efficacy of macrocyclic lactones is very limited. It was pointed out that, in both roe deer and red deer, the application of a single oral dose of 0.4 mg/kg b.w. of ivermectin achieved 100% efficacy against Oestrinae and Hypodermatinae [65]. In another investigation, ivermectin was added to the feed of roe deer (150 mg/kg) during the winter months for 8 consecutive years; although it was estimated that the daily feed intake would be 0.5 kg/10 kg of b.w. per day, this treatment was not totally effective, since average figures of 7.3 *C. stimulator* larvae/animal were observed at necropsy [34].

It is worth noting that the excessive, repeated and inappropriate use of endectocides has favored the development of nematode resistance, especially in sheep and, less frequently, in equids and cattle [66]. In this respect, resistance to abamectin and ivermectin has been currently described in houseflies [67] and ticks [68], respectively. Although oestrid resistance to macrocyclic lactones has not yet been reported, there is no reason to think that its development is not possible [69].

Due to the difficulty involved in the management of wildlife, the best alternative for the administration of drugs is through feed available to animals at feeders. This implies that a significant number of animals can ingest lower doses than recommended, with the risk of developing resistance to these drugs. In addition, macrocyclic lactones are partially metabolized in the rumen [70], so for their effectiveness to be similar to their parenteral administration, a dose 3–4 times higher must be administered.

The use of macrocyclic lactones presents also some drawbacks related to environmental and public health problems (residues in meat). In this regard, the European Agency for the Evaluation of Medicinal Products [62] reported, in 1998, that available data on the metabolism of ivermectin in cervids are still limited. However, it concludes that deer fat contains more residues of macrocyclic lactones than that of cattle, even though ivermectin elimination seems faster in deer than in cattle. In addition, it is assumed that the marker of the presence of residues in cervids is the same compound as in other species (22,23-Dihydroavermectin B1a) and the maximum residue limits (MRLs) of 20 μg/kg in muscle, 100 μg/kg in fat, 50 μg/kg in liver and 20 μg/kg in kidney are established.

The negative impact of macrocyclic lactones on the environment is well known, especially for dung fauna and dung degradation [70]. After oral administration, non-metabolized drug can be detected in feces, maintaining their ecotoxicity [70]. Moreover, oral and pour-on applications of macrocyclic lactones have greater environmental consequences, as they require higher doses than parenteral formulations [71].

Roe deer populations are currently abundant in Europe. Although this wild ungulate has been almost extinct in parts of southern Europe because of habitat loss and overhunting in the first half of the XX century, its numbers started to recover 20–40 years ago and they are still increasing [72]. Nevertheless, high roe deer density favors parasite transmission and dispersion and causes a high degree of social stress that enhances the negative effect of parasitic infections [72]. For these reasons, management strategies focused on lowering/controlling roe deer densities should be accomplished for limiting the impact of cephenemyiosis and other pathogens on the health of the European roe deer.

Finally, several roe deer reintroduction/restocking programs carried out for decades all over Europe have favored the spread of *C. stimulator* as it has occurred in Spain [26]. Translocations from *Cephenemyia* endemic areas should be strictly controlled and conducted after an effective treatment to avoid the extension of this parasite to unexposed areas.

## 9. Conclusions

Cephenemyiosis is a myiasis that in the last decade is expanding its distribution along southernmost and northernmost range margins of the European roe deer. The severity of the disease is related to the larval burden, so that a moderate number of larvae (>30 L3) is generally well tolerated by the host. Treating roe deer against *C. stimulator* is theoretically possible, since some drugs, especially macrocyclic lactones, have been proven to be effective against oestrids However, their use is controversial in wildlife; therefore, alternative control measures, such as management strategies focused on lowering/controlling roe deer densities are needed.

## Figures and Tables

**Table 1:** *Cephenemyia* species, distribution and main hosts.

Species	Distribution	Hosts
*C. trompe* (Modeer, 1786)	Neartic/Paleartic	deer, moose, reindeer/caribou
*C. ulrichii* (Brauer, 1863)	Paleartic	moose
*C. auribarbis* (Meigen, 1824)	Paleartic	red deer
*C. stimulator* (Hunter, 1916)	Paleartic	roe deer
*C. phobifer* (Clark, 1815)	Neartic	white-tailed deer
*C. apicata* (Bennett & Sabrosky, 1962)	Neartic	mule deer
*C. jellisoni* (Townsend, 1941)	Neartic	mule deer, white-tailed deer, moose, elk
*C. pratti* (Hunter, 1916)	Neartic	mule deer, white-tailed deer

**Table 2:** Prevalence and intensity of infestation by *C. stimulator* in roe deer from Europe.

Country	Prevalence	Intensity	Method	References
Hungary	70.8	12	Necropsy	[50,51]
	34.8–35.2	9.8–8.8	Necropsy	[52]
	11.1–76.9	3.9–19	Necropsy	[20]
	17.3–22.9	15.3–25.4	Necropsy	[53,54]
Czech Republic	60–90	-	Necropsy	[55]
	11.1–25.6	7.7	Necropsy	[18]
	44	13	Necropsy	[56]
	16.1–42.9	6–11	Necropsy	[37]
Poland	13	1–10	Necropsy	[57]
Germany	49	3–11	Necropsy	[58]
Croatia	27	-	Necropsy	[59]
France	32–43.2	-	iELISA	[49]
Spain	40	11–49	Necropsy	[46]
	31.6	19.7 ± 21	Necropsy	[31]
	36–60	-	iELISA	[31]
	23.8	-	iELISA	[28]
	43.2	16.9 ± 22.47	Necropsy	[13]

## Data Availability

Not applicable.
